# ChatGPT and Artificial Intelligence in Transplantation Research: Is It Always Correct?

**DOI:** 10.7759/cureus.42150

**Published:** 2023-07-19

**Authors:** Badi Rawashdeh, Joohyun Kim, Saif Aldeen AlRyalat, Raj Prasad, Matthew Cooper

**Affiliations:** 1 Transplant Surgery, Medical College of Wisconsin, Milwaukee, USA; 2 Opthalmology, The University of Jordan, Amman, JOR

**Keywords:** research, chatgpt, kidney transplantation, machine learning, artificial intelligence

## Abstract

Introduction: ChatGPT (OpenAI, San Francisco, California, United States) is a chatbot powered by language-based artificial intelligence (AI). It generates text based on the information provided by users. It is currently being evaluated in medical research, publishing, and healthcare. However, there has been no prior study on the evaluation of its ability to help in kidney transplant research. This feasibility study aimed to evaluate the application and accuracy of ChatGPT in the field of kidney transplantation.

Methods: On two separate dates, February 21 and March 2, 2023, ChatGPT 3.5 was questioned regarding the medical treatment of kidney transplants and related scientific facts. The responses provided by the chatbot were compiled, and a panel of two specialists reviewed the correctness of each answer.

Results: We demonstrated that ChatGPT possessed substantial general knowledge of kidney transplantation; however, they lacked sufficient information and had inaccurate information that necessitates a deeper understanding of the topic. Moreover, ChatGPT failed to provide references for any of the scientific data it provided regarding kidney transplants, and when requested for references, it provided inaccurate ones.

Conclusion: The results of this short feasibility study indicate that ChatGPT may have the ability to assist in data collecting when a particular query is posed. However, caution should be exercised and it should not be used in isolation as a supplement to research or decisions regarding healthcare because there are still challenges with data accuracy and missing information.

## Introduction

Artificial intelligence (AI) refers to any computer system that has been designed to learn and mimic the human brain [1]. Machine learning (ML) is a form of AI that uses large amounts of data to find various patterns in it [2]. ML is a type of AI that uses significant amounts of data to discover various patterns [3,4].

ChatGPT (Chat Generative Pre-trained Transformer) is a type of ML system that can produce natural-sounding, sophisticated, realistic, and human-like text [5,6]. Its base model, GPT-3, was trained on articles, websites, books, and written conversations, but a process of fine-tuning (including optimization for dialogue) enables ChatGPT to respond to prompts in a conversational way [7]. It learns on its own from data and is trained on a massive database of text to create sophisticated and apparently intelligent writing [6]. ChatGPT is a fast, free, and easy-to-use AI chatbot platform that was introduced in November 2022 by the AI research firm OpenAI, based in San Francisco, California, United States. ChatGPT is bound to hugely impact many industries, including entertainment, finance, news, and healthcare, and medical scientific research and writing are no exceptions [6,8].

ChatGPT is able to provide clear and convincing written responses to queries from anyone, including patients, on a wide range of medical topics [5]. In addition to passing several medical exams, including the United States Medical Licensing Exam (USMLE) [9], ChatGPT was also able to help in writing scientific manuscripts [10,11] and was even approved as a reference in medical journals [9,12]. However, many concerns have been voiced about the accuracy of the information given when ChatGPT is used in medical research [13,14], and it is currently unknown whether or not this model will be accurate and reliable when applied specifically to the field of kidney transplant research.

ChatGPT has been studied for its potential to improve the efficiency and cost-effectiveness of clinical practices [15]. ChatGPT demonstrated transformative potential in healthcare practice by improving diagnostics, disease risk, and outcome prediction, among other areas of translational research [16]. The reasonable accuracy with which ChatGPT predicted the imaging procedures required for cancer screening suggests that it may have useful applications in radiology decision-making [16,17]. Using ChatGPT in healthcare settings also offers the potential to advance customized medicine and boost health literacy by making vital health information more accessible to and understood by the public [15].

The usage of ChatGPT in healthcare settings has, however, been met with some criticism [17]. Ethical concerns, such as the risk of bias and transparency issues, emerged as significant concerns. In addition, the production of inaccurate content can have serious adverse impacts on healthcare; consequently, this legitimate concern should be carefully considered in healthcare practice [15].

Therefore, this feasibility study aimed to evaluate the application and accuracy of the research assistance provided by ChatGPT for the transplantation field. We formulated a wide-ranging discussion with ChatGPT on kidney transplantation-related topics and evaluated ChatGPT's ability to produce accurate scientific writing.

## Materials and methods

Study design

On February 21, 2023, and March 2, 2021, we conducted a brief investigation using the publicly accessible website https://chat.openai.com/chat to evaluate the potential use of ChatGPT 3.5 in medical scenarios related to kidney transplantation. We first submitted a number of questions to ChatGPT on a variety of topics pertaining to kidney transplantation (Table 1). Second, we evaluated ChatGPT's ability to produce correct scientific writing on topics related to kidney transplantation. Third, we evaluated its ability to summarize specific articles and provide references on a wide range of kidney transplant-related topics. Two experts independently assessed the accuracy of the responses provided by ChatGPT. Clarity of responses, scientific accuracy of content, and conciseness were parameters that were used to evaluate responses to questions posed on the ChatGPT. The Institutional Review Board's approval and informed consent were not necessary for this study because it did not utilize patient data.

**Table 1:** Questions used to assess the scientific content of ChatGPT* regarding various kidney transplantation-related topics. *OpenAI, San Francisco, California, United States HCV: hepatitis C virus

Number	Questions
1	What is the definition of delayed graft function?
2	What is the role of eculizumab in delayed graft function?
3	Can someone receive a kidney from a donor who has HCV?
4	What is the success rate and duration of treatment of direct-acting antiviral (DAA) drugs in treating HCV after a kidney transplant?

## Results

ChatGPT had a thorough understanding of kidney transplantation topics in general, but it was unable to provide adequate detail or accurate answers to questions that required more in-depth knowledge of the subject. ChatGPT responded in scientifically sound writing, but the data it came up with was a mix of factual evidence and entirely fabricated details. ChatGPT's responses on two different days were nearly identical, and we didn't observe any difference in scientific data or clarity on either day for each question asked.

The responses provided by ChatGPT to questions about general kidney transplant-related topics were well-written, clear, and convincing, as well as generally accurate, particularly with regard to the definitions of common terms in the kidney transplant field. None of ChatGPT's answers to assessment questions on clinical situations and treatment involving kidney transplant subjects at the level of providers and researchers, which requires a deeper understanding of the field, were error-free. Furthermore, when asked for references, ChatGPT provided inaccurate ones and still failed to provide a reliable source for the scientific data it presented on kidney transplantation (Table 2).

**Table 2:** ChatGPT* responses to researcher queries about various kidney transplantation-related topics *OpenAI, San Francisco, California, United States HCV: hepatitis C virus; DGF: delayed graft function

	Question	ChatGPT response on trial 1	ChatGPT response on trial 2
1	What is the definition of delayed graft function?	Delayed graft function (DGF) is a common complication that can occur following kidney transplantation. It is defined as a period of slow or absent function of the transplanted kidney immediately after transplantation. DGF is usually diagnosed when there is a need for dialysis within the first week after transplantation, although the exact definition can vary slightly depending on the transplant center. DGF occurs due to a number of factors, including prolonged cold ischemia time, poor organ quality, and damage to the kidney during the transplantation process. The condition is more common in kidneys from deceased donors, particularly those that have been subject to prolonged ischemic times, and in recipients who have pre-existing medical conditions that can impair kidney function. Although DGF is a serious complication, it is usually reversible with appropriate treatment, and most patients will eventually recover normal kidney function. The treatment of DGF typically involves close monitoring of the patient's kidney function, optimization of immunosuppressive therapy, and supportive measures such as dialysis or fluid management as needed.	Delayed graft function (DGF) is a common complication that can occur after kidney transplantation. It is defined as a condition in which the transplanted kidney does not function immediately or as expected after the transplant surgery. DGF is characterized by a slow or inadequate recovery of kidney function, and it can result in a prolonged need for dialysis after transplantation. The condition can be caused by a variety of factors, including: Prolonged cold ischemia time: the amount of time the kidney is without blood flow and oxygen before transplantation; Reperfusion injury: damage to the kidney tissue caused by the restoration of blood flow after transplantation; Donor factors: such as age, hypertension, or preexisting kidney disease; Recipient factors: such as diabetes, obesity, or a history of previous kidney transplants; Immunological factors: such as the presence of donor-specific antibodies. The diagnosis of DGF is typically made based on laboratory values such as serum creatinine levels and urine output, as well as clinical symptoms such as fluid overload or electrolyte imbalances. DGF is usually treated with supportive measures such as dialysis, careful monitoring of fluid and electrolyte balance, and close management of immunosuppressive medications to prevent rejection of the transplanted kidney. With appropriate management, most cases of DGF resolve over time, and the transplanted kidney eventually regains normal function. However, in some cases, DGF can lead to more serious complications, such as primary nonfunction or chronic rejection, which can result in the need for re-transplantation or return to dialysis.
2	what is the role of eculizumab in delayed graft function?	Eculizumab is a medication that is used to treat certain types of complement-mediated diseases, such as atypical hemolytic uremic syndrome (aHUS) and paroxysmal nocturnal hemoglobinuria (PNH). While eculizumab is not a standard treatment for DGF, there is some evidence to suggest that it may be helpful in certain cases. DGF can occur due to a variety of factors, including immune-mediated injury to the transplanted kidney. Complement-mediated injury is one mechanism by which the immune system can damage the transplanted kidney. Eculizumab is a medication that inhibits the complement system, which may reduce the risk of complement-mediated injury to the transplanted kidney. Several studies have explored the use of eculizumab in the treatment of DGF. While the results have been mixed, some studies have suggested that Eculizumab may be beneficial in reducing the risk of DGF and improving kidney function in some patients. However, more research is needed to determine the optimal dosing, timing, and duration of eculizumab therapy for DGF. It is important to note that eculizumab is a specialized medication that is typically used in highly specialized medical centers, and its use in the treatment of DGF should be carefully considered by a team of experienced transplant professionals. The use of eculizumab for DGF should be individualized based on the specific needs and medical history of the patient.	Eculizumab is a medication that targets a protein in the complement system called C5, which is involved in inflammation and immune system activation. The use of eculizumab has been investigated as a potential treatment for DGF after kidney transplantation. Several studies have suggested that eculizumab may be effective in improving kidney function and reducing the incidence of DGF after transplantation. The exact mechanism by which eculizumab improves kidney function in DGF is not fully understood, but it is thought to be related to its ability to block the activation of the complement system and reduce inflammation. One study published in the New England Journal of Medicine in 2019 found that treatment with eculizumab significantly reduced the incidence of DGF after kidney transplantation. In the study, patients who received eculizumab had a lower rate of DGF compared to those who received a placebo. The study also found that eculizumab treatment was associated with improved kidney function, reduced inflammation, and a lower incidence of acute rejection. However, the use of eculizumab for the treatment of DGF after kidney transplantation is not yet widely accepted or recommended, and further studies are needed to determine its safety and efficacy. Eculizumab is an expensive medication that requires careful monitoring, and it may have potential side effects, such as increased risk of infections. In summary, while eculizumab has shown promising results in some studies, its use in the treatment of DGF after kidney transplantation is still being evaluated, and it should only be used in carefully selected patients under close medical supervision.
3	Can someone receive a kidney from a donor who has HCV?	Yes, it is possible for someone to receive a kidney from a donor who has HCV. However, in the past, people with HCV were generally excluded as potential organ donors due to the risk of transmitting the virus to the recipient. In recent years, advances in medical treatments have led to new medications that can cure HCV, making it possible to use organs from donors with HCV. These treatments are highly effective and have greatly reduced the risk of transmitting HCV to transplant recipients. Transplant centers now routinely evaluate the HCV status of potential donors and recipients, and they may consider using organs from donors with HCV for recipients who also have HCV or for recipients who do not have HCV but who are willing to receive an HCV-positive organ. It's worth noting that the decision to use organs from HCV-positive donors is made on a case-by-case basis and depends on several factors, including the HCV genotype and viral load of the donor, the HCV status and medical condition of the recipient, and the availability of other organs. Transplant centers carefully evaluate all potential donors and recipients to ensure the best possible outcome for the recipient.	Yes, it is possible for someone to receive a kidney from a donor who has HCV. In fact, in recent years, there has been a significant increase in the use of HCV-positive organs for transplantation, especially in the context of the current opioid epidemic, which has led to an increase in the number of potential organ donors with HCV. The reason why HCV-positive organs are being used is that effective antiviral treatments are now available that can cure HCV infection in the recipient, preventing the virus from causing liver damage or other health problems. Moreover, studies have shown that the use of HCV-positive kidneys for transplant can result in good outcomes, with similar long-term graft survival and patient survival rates compared to organs from HCV-negative donors. However, not all transplant centers are currently using HCV-positive organs, and the decision to use such organs is made on a case-by-case basis. Factors that may influence this decision include the HCV genotype and viral load of the donor, the HCV status and medical condition of the recipient, and the availability of other organs. It's important to note that recipients who receive an HCV-positive organ will need to undergo antiviral treatment to cure the HCV infection.
4	What is the success rate and duration of treatment of direct-acting antiviral (DAA) drugs in treating HCV after a kidney transplant?	The success rate of direct-acting antiviral (DAA) drugs in treating HCV after a kidney transplant is very high, with cure rates of up to 95-100%. The exact success rate can vary depending on factors such as the patient's HCV genotype, the degree of liver damage, and whether the patient has previously undergone treatment for HCV. The duration of treatment with DAA drugs for HCV after a kidney transplant can vary depending on the specific medication used and other factors. Typically, treatment can last anywhere from 8 to 24 weeks, with most patients receiving treatment for 12 weeks. Patients who have previously undergone HCV treatment or who have certain other risk factors may require longer treatment durations. It's important to note that the decision to treat HCV after a kidney transplant should be made on a case-by-case basis by a healthcare professional, taking into account individual patient factors such as the severity of the infection, the patient's overall health, and the potential risks and benefits of treatment. Regular monitoring and follow-up are also important after HCV treatment to ensure that the infection does not return and that the patient's overall health is maintained.	The success rate of direct-acting antiviral (DAA) drugs in treating HCV after a kidney transplant is generally high, with cure rates ranging from 90% to 100%. Studies have shown that DAAs are effective in treating HCV in kidney transplant recipients, with similar rates of sustained virologic response (SVR), which is defined as undetectable HCV RNA in the blood 12 weeks after the end of treatment, compared to HCV treatment in non-transplant patients. The duration of treatment for HCV after kidney transplantation typically ranges from 8 to 12 weeks, depending on the genotype of the HCV virus and the degree of liver damage. However, in some cases, treatment may need to be extended beyond 12 weeks. Treatment with DAAs is generally well-tolerated by kidney transplant recipients, with a low risk of serious side effects or drug interactions with other medications. Successful treatment of HCV with DAAs has been shown to improve kidney function and increase graft survival rates in kidney transplant recipients. Moreover, successful treatment of HCV after kidney transplantation can also prevent the transmission of the virus to other individuals. It's important to note that the decision to treat HCV in kidney transplant recipients with DAAs should be individualized based on several factors, including the patient's overall health, the degree of liver damage, and the risk of drug interactions with other medications. Therefore, patients should discuss the potential benefits and risks of HCV treatment with their healthcare providers.

## Discussion

The results of our research clearly demonstrated that ChatGPT can provide convincing, well-written responses, but the material it delivered regarding kidney transplantation is a mixture of true facts and wholly incorrect ones, potentially resulting in the development of false information. This raises questions regarding the reliability, dependability, and credibility of utilizing a large language model (LLM) such as ChatGPT in scientific writing on subjects related to kidney transplants.

Our findings show that ChatGPT can define general terms appropriately, similar to prior studies examining its capacity for accurately addressing general public health subjects [16]. However, when we started to ask ChatGPT a higher level of medical questions that necessitated deeper knowledge of the subject, ChatGPT’s responses were not accurate and not supported by evidence; this shortcoming of ChaGPT has been cited by other studies [15,18].

AI systems offer tremendous potential for enhancing medical care and health outcomes [1]. ChatGPT, as a language-based AI, may have a big influence on how researchers in the medical field approach their work in the future, as they have already used ChatGPT and other LLMs to write essays and presentations, summarize the literature, draft and improve papers, and even conduct statistical analyses [10,11]. 

ChatGPT is a transformer-based model; these models include two main steps to process text data: pre-training and fine-tuning [19]. Pre-training includes feeding the models massive and diverse amounts of data and asking them to predict the next word in each sentence [19,20]. When training is complete, the model can be further fine-tuned (customized) to perform specific tasks, such as conversing with users, answering questions, or even specializing in a certain domain. This process of pre-training and fine-tuning allows GPT language models to understand patterns and statistical relationships between words in the text. During inference, transformers tokenize text data into discrete units and generate probability distributions for the possible next tokens, thus generating logical and human-like responses [19,20].

ChatGPT has generated controversy and concerns in the medical research field because it is one of the first models that can convincingly converse with users in English and other languages, and on a variety of topics [12,14]. ChatGPT and other LLMs generate text that is persuasive but frequently scientifically incorrect, so their use can distort scientific facts and propagate misinformation [8,21]. The use of conversational AI for specialized research might result in errors, bias, and plagiarism [16].

We gave ChatGPT a set of questions about kidney transplant that required a thorough knowledge of the literature and discovered that it frequently produced false and deceptive text. We tested ChatGPT in different areas; for example, we asked the chatbot if someone with end-stage kidney disease could receive a kidney from a donor who had a hepatitis C virus (HCV) infection. ChatGPT was able to provide an accurate response and added that the new direct antiviral agents (DAA) made this a feasible and safe practice. When we inquired about the treatment, the response was accurate in terms of duration and efficacy; however, none of the responses were supported by references and contained some incorrect statements, such as the claim that DAA treatment would improve long-term outcomes after transplantation (Table 2).

Then, we requested that ChatGPT provide us with information on the subject of delayed graft function (DGF) and cite sources. ChatGPT provided accurate basic information about DGF; however, it specifically referred to one study published in the New England Journal of Medicine in 2019, which we couldn't find despite our thorough search on PubMed and Web of Science databases, and claimed that this study found that treatment with eculizumab significantly reduced the incidence of DGF after kidney transplantation. ChatGPT also stated that the study found that patients who were administered eculizumab had a lower incidence of DGF than those who were given a placebo. According to ChatGPT, the study additionally showed that eculizumab treatment was associated with improved kidney function, decreased inflammation, and a lower incidence of acute rejection. However, evidence from randomized controlled trials demonstrates that, while peritransplant eculizumab can be safely administered to recipients of deceased donor kidney transplants, it has no efficacy in preventing or minimizing the development of DGF [22].

**Figure 1:**
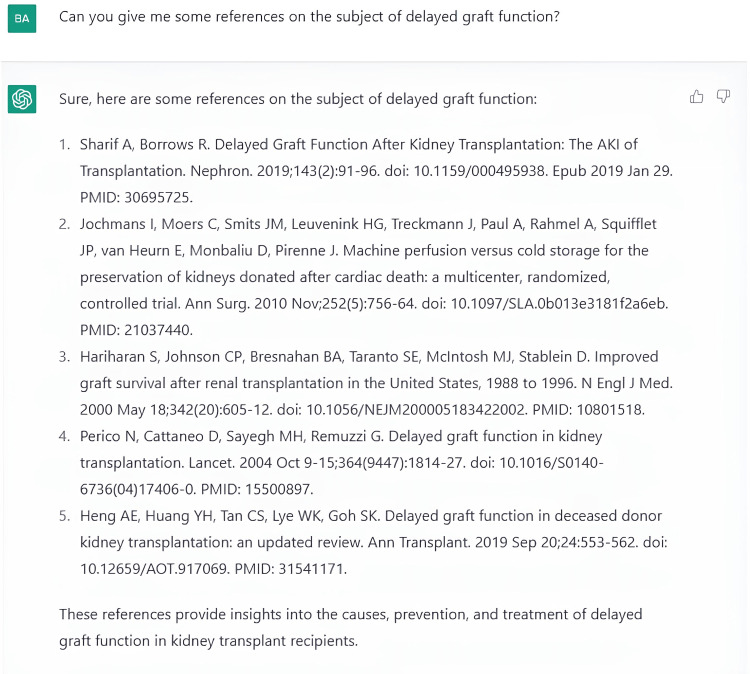
ChatGPT response to an inquiry for references on the topic of delayed graft function

It provided five references, yet only one of the article names was accurate, two of the PubMed IDs (PMIDs) were for unrelated papers, and three references weren't even listed on PubMed. For example, the citation that ChatGPT provided is "Jochmans I, Moers C, Smits JM, Leuvenink HG, Treckmann J, Paul A, Rahmel A, Squifflet JP, van Heurn E, Monbaliu D, Pirenne J. Machine perfusion versus cold storage for the preservation of kidneys donated after cardiac death: a multicenter, randomized, controlled trial Ann Surg. 2010 Nov; 252(5):756-64. Doi: 10.1097/SLA.0b013e3181f2a6eb. PMID: 21037440", When we searched using the PMID provided, the resulting paper was entirely different and in a different field (Figure 1).

Next, we asked that ChatGPT summarize an article we had published on the use of the robotic technique on morbidly obese patients [23]. ChatGPT summarized the article's findings but provided an incorrect number of patients included in the study. Furthermore, we asked ChatGPT to summarize an article we published titled "Impact of COVID-19 on abdominal organ transplantation: A bibliometric analysis" [24]. ChatGPT responded with the wrong number of articles studied in our bibliometric study as well as the wrong timeframe, and it falsely claimed that we as the authors suggested that "future research in this area should focus on the long-term effects of COVID-19 on transplant outcomes and the development of effective strategies for managing transplant recipients", a subject that was not discussed or included in our paper.

In view of the findings from previous studies that examined the feasibility of using ChatGPT in clinical and research settings, it is clear that there is a need to raise awareness of the potential advantages and disadvantages of utilizing AI-based LLMs in healthcare [16,25].

ChatGPT provides easy access to general information on transplantation, serving as a resource for public awareness. However, it is important to exercise caution as the information can be both informative and potentially misleading. Our research clearly demonstrated that ChatGPT, despite its apparent ability to generate convincing scientific essays, produces a mixture of legitimate and completely fabricated information in the field of kidney transplant. It is therefore questionable whether LLM like ChatGPT should be used in scientific research. We advocate for a shift in policy and practice regarding the review of scientific papers for publication in journals and presented at medical conferences so that the highest possible standards can be maintained. We also call for transparent disclosure of the use of these technologies and the incorporation of AI output detectors into the editing process.

This article provides the first general evaluation of ChatGPT's utility in addressing issues around kidney transplantation. The current review's findings should be interpreted cautiously due to its limitations, such as the small number of questions and the fact that the ChatGPT wasn't asked questions on every aspect of kidney transplantation. The aforementioned findings cannot be directly applied to other topics or medical disciplines, as chatbots will likely continue to evolve rapidly in response to user feedback. A subsequent experiment with the same items may produce differing results.

## Conclusions

This evaluation has demonstrated that ChatGPT is able to define terms precisely and provide general information about different topics related to kidney transplant. However, in our study, ChatGPT was unable to provide references and its responses contained inaccurate data, showing a lack of in-depth understanding of the subject. The findings of this small feasibility study demonstrate that ChatGPT has the ability to assist with data collection at this time, including publications when given a particular question. However, there are still issues with accuracy or missed data, so ChatGPT should be used with caution and not by itself or as a supplement to research or medical decisions.
